# Comparison of the Anti-SARS-CoV-2 Surrogate Neutralization Assays by TECOmedical and DiaPROPH-Med with Samples from Vaccinated and Infected Individuals

**DOI:** 10.3390/v14020315

**Published:** 2022-02-03

**Authors:** Lennart Münsterkötter, Moritz Maximilian Hollstein, Andreas Hahn, Andrea Kröger, Moritz Schnelle, Luise Erpenbeck, Uwe Groß, Hagen Frickmann, Andreas Erich Zautner

**Affiliations:** 1 Institute for Medical Microbiology, University Medical Center Göttingen, 37075 Göttingen, Germany; l.muensterkoetter@stud.uni-goettingen.de (L.M.); ugross@gwdg.de (U.G.); 2 Department of Dermatology, Venereology and Allergology, University Medical Centre Göttingen, 37075 Göttingen, Germany; moritz.hollstein@med.uni-goettingen.de (M.M.H.); luise.erpenbeck@ukmuenster.de (L.E.); 3 Institute for Medical Microbiology, Virology and Hygiene, University Medicine Rostock, 18057 Rostock, Germany; andreas.hahn@uni-rostock.de (A.H.); frickmann@bnitm.de (H.F.); 4 Institute of Medical Microbiology and Hospital Hygiene, Medical Faculty, Otto-von-Guericke University Magdeburg, 39120 Magdeburg, Germany; andrea.kroeger@med.ovgu.de; 5 Research Group Innate Immunity and Infection, Helmholtz Centre for Infection Research, 38124 Braunschweig, Germany; 6 Institute for Clinical Chemistry, University Medical Center Göttingen, 37075 Göttingen, Germany; moritz.schnelle@med.uni-goettingen.de; 7 Department of Dermatology, University of Münster, 48149 Münster, Germany; 8 Department of Microbiology and Hygiene, Bundeswehr Hospital Hamburg, 20359 Hamburg, Germany

**Keywords:** SARS-CoV-2, COVID-19, serology, neutralization, test comparison, vaccination

## Abstract

Anti-SARS-CoV-2-specific serological responses are a topic of ongoing evaluation studies. In the study presented here, the anti-SARS-CoV-2 surrogate neutralization assays by TECOmedical and DiaPROPH -Med were assessed in a head-to-head comparison with serum samples of individuals after vaccination as well as after previous infection with SARS-CoV-2. In case of discordant results, a cell culture-based neutralization assay was applied as a reference standard. The TECOmedical assay showed sensitivity and specificity of 100% and 61.3%, respectively, the DiaPROPH-Med assay 95.0% and 48.4%, respectively. As a side finding of the study, differences in the likelihood of expressing neutralizing antibodies could be shown for different exposition types. So, 60 of 81 (74.07%) of the samples with only one vaccination showed an expression of neutralizing antibodies in contrast to 85.71% (60 of 70 samples) of the samples with two vaccinations and 100% (40 of 40) of the samples from previously infected individuals. In conclusion, the both assays showed results similar to previous assessments. While the measured diagnostic accuracy of both assays requires further technical improvement of this diagnostic approach, as the calculated specificity values of 61.3% and 48.4%, respectively, appear acceptable for diagnostic use only in populations with a high percentage of positive subjects, but not at expectedly low positivity rates.

## 1. Introduction

Although protective immunity is considered a key element in order to overcome the COVID-19 pandemic, vaccine-induced immune-protection is not associated with sterile immunity and is prone to be undermined by virus variants [1]. Serological screening assays were rapidly developed along with the global spread of SARS-CoV-2 [2,3]. However, it soon became obvious that the reliability of such diagnostic strategies depends on various factors including uneven geographic distributions of cross-reacting antibodies [4].

Further, it was clear that the functionality of adaptive immunological components is more important than just sole quantity [5]. In line with this, it became evident that a diagnostic focus should be on the neutralizing the potency of detected anti-SARS-CoV-2-antibodies [6,7]. Correlations between the severity of clinical courses of COVID-19 disease and neutralizing antibody titers could be shown [8,9,10,11].

To circumvent the need for laborious and time-consuming tissue culture infecting dose-(TCID-) assays for the assessment of the neutralizing potency of anti-SARS-CoV-2-antibodies, alternative diagnostic strategies were implemented and tested. Those attempts comprised correlations of total and neutralizing anti-SARS-CoV-2-antibodies [12,13,14,15]. In addition, however, specific antigenic target structures were identified in order to develop surrogate neutralization assays for testing for antibodies specific to those critical epitopes [16,17].

In the meantime, numerous cellular and acellular surrogate neutralization assays have been introduced and evaluated [18,19,20,21,22,23,24,25,26,27,28,29,30,31,32,33,34,35,36,37,38,39,40,41,42,43,44,45]. Such evaluations of both neutralizing and overall anti-SARS-CoV-2-antibodies comprised both natural infections and post-vaccination states [46,47,48,49,50]. Next to neutralizing potency, antibody avidity was included in the long-term assessments as well [51].

In the study presented here, two surrogate neutralization assays, i.e., the TECO SARS-CoV-2 Neutralization Antibody Assay (TECOmedical AG, Sissach, Switzerland), for which previous assessments had suggested imperfect sensitivity and specificity in the 90% range [24,25], and the DIA-SARS-CoV-2-nAb (DiaPROPH Med, Kiev, Ukraine) surrogate neutralization assay, were compared in a head-to-head assessment with serum samples from vaccinated and infected individuals. The choice of the assays was based on the fact that first evaluation results for the TECOmedical assay were already available [24,25] allowing at least a rough estimation of the credibility of the results of the present study, while no peer-reviewed published data on the DiaPROPH assay were available so far. By applying this approach, this communication intends to contribute to the puzzle of diagnostic accuracy assessment of commercially available serological SARS-CoV-2 assays. Quantitative measurement of overall-anti-SARS-CoV-2 antibodies and avidity testing were comparatively added and contradicting results in the surrogate neutralization assays were finally decided by a cell culture-based neutralization assay.

## 2. Materials and Methods

### 2.1. Samples Included in the Assessment

For the assessment, 81 serum samples were obtained after the first vaccination against SARS-CoV-2, 70 serum samples after the second vaccination against SARS-CoV-2 and 40 samples from individuals with a history of natural infections with the SARS-CoV-2 virus. For the vaccinees, who were participants in the COV-ADAPT study [52], anti-SARS-CoV-2-NCP-ELISA (IgG) (Euroimmun, Lübeck, Germany) excluded anti-nucleocapsid-IgGs (NCP) to rule out infection with SARS-CoV-2 before vaccination. Negative results were a prerequisite for being included in the assessment. Vaccinations had been performed with various combinations of Vaxzevria (AstraZeneca, Cambridge, UK) or Comirnaty (Biontech, Mainz, Germany). Infections that occurred during a period when the Wuhan wild type variant was prevalent had been ensured by real-time PCR. Based on the various combinations of possible expositions, three different exposition types, i.e., natural infection, exposure to one vaccination and exposure to two vaccinations, were defined.

### 2.2. Performed Diagnostic Assessments

**Figure 1:**
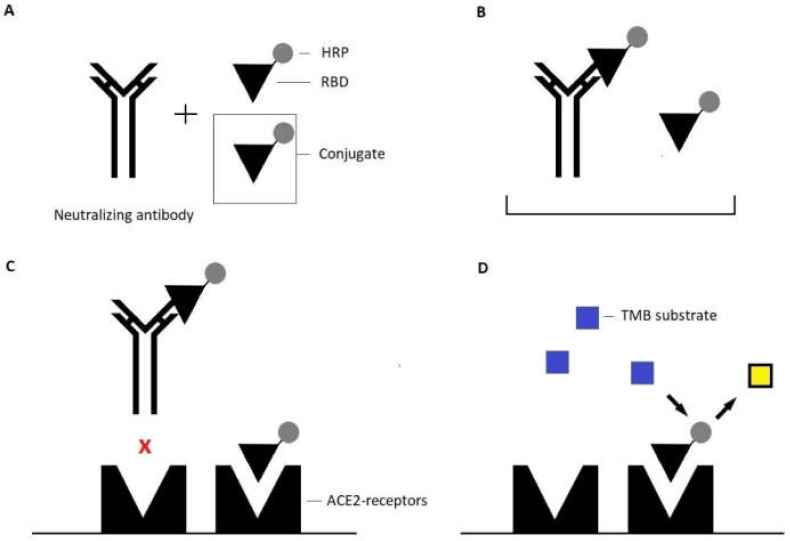
Principle of the TECOmedical SARS-CoV-2 Neutralization Antibody ELISA. (**A**) Serum samples and conjugate (HRP-labelled SARS-CoV-2-SP-RBD) are premixed. (**B**) During incubation, neutralizing antibodies (anti-SARS-CoV-2-RBD-IgG) and the conjugate form antibody–antigen complexes. (**C**) The mixture is added to the solid phase; preformed complexes are unable to interact with ACE2-receptors; unbound conjugate can interact freely. (**D**) Tetramethylbenzide substrate is added and reacts with HRP; the measured optical density is inversely correlated with the concentration of neutralizing antibodies. Yellow square: color change after enzymatic reaction.

All sera were comparatively assessed for neutralizing anti-SARS-CoV-2 antibodies using the surrogate neutralization assays TECO SARS-CoV-2 Neutralization Antibody Assay (TECOmedical AG, Sissach, Switzerland) and DIA-SARS-CoV-2-nAb (DiaPROPH Med, Kiev, Ukraine; distributed by AlphaScience GmbH, Riedstadt, Germany) as described by the manufacturer using the DSX Automated ELISA System (Thermo Labsystems, Chantilly, VA, USA). Both assays are competitive ELISAs. In detail, during the TECO SARS-CoV-2 Neutralization Assay (later also referred to as the “TECOmedical surrogate neutralization assay”), anti-SARS-CoV-2-IgG present in tested sera are bound to a conjugate during preincubation (Figure 1). The conjugate consists of a HRP-(horseradish peroxidase-) labelled receptor binding domain of the viruses’ spike protein. Formed complexes block the conjugate’s interaction with ACE2-receptors on the solid phase. Added tetramethylbenzide (TMB) substrate then reacts with the remaining conjugate that successfully interacted with the mentioned receptors. When read at 450/620 nm, the sample’s OD is inversely correlated with the concentration of present neutralizing antibodies.

**Figure 2:**
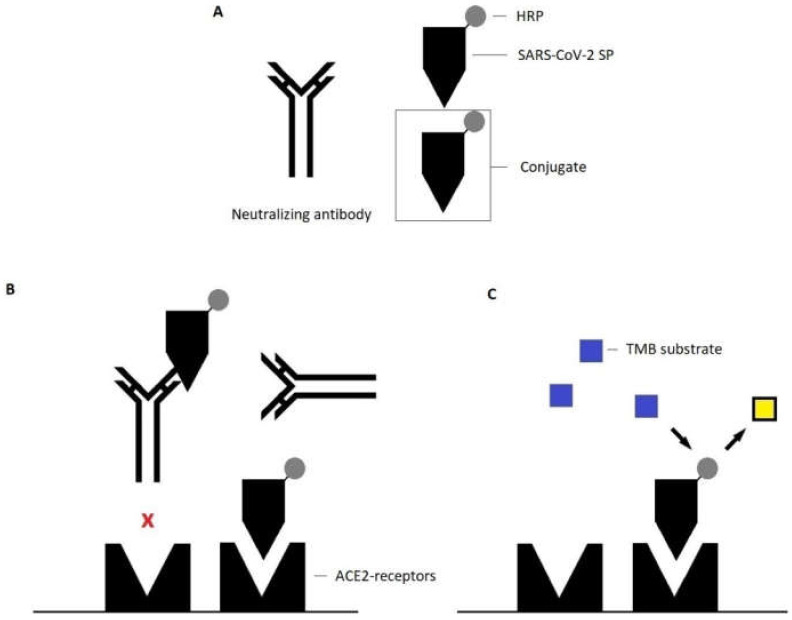
Principle of the DIA-SARS-CoV-2-nAb assay. (**A**) Serum samples and conjugate (HRP-labelled recombinant SARS-CoV-2-spike-protein) are simultaneously added to the solid phase. (**B**) Present neutralizing antibodies (anti-SARS-CoV-2-RBD-IgG) are bound to the conjugate and consequently block the interaction between the receptor-binding domain of the spike protein and recombinant human ACE-2-receptors on the solid phase. (**C**) Tetramethylbenzide substrate is added and reacts with HRP; the measured optical density is inversely correlated with the concentration of neutralizing antibodies in the sample. Yellow square: color change after enzymatic reaction.

In contrast to that, the DIA-SARS-CoV-2-nAb (later also referred to as “DiaPROPH-Med surrogate neutralization assay) makes use of an HRP-labelled recombinant human spike protein as a conjugate, which directly competes with neutralizing antibodies in the test wells (Figure 2). The latter prevents the conjugate’s interaction with recombinant human ACE2-receptors. TMB substrate is again used to detect HRP-labelled spike protein that successfully interacted with the solid phase. When read at 450/620 nm, the sample’s OD is inversely correlated with the concentration of the present neutralizing antibodies.

In addition, all samples were subjected to an avidity assessment of anti-SARS-CoV-2 antibodies using the DIA-SARS-CoV-2-S-IgG-av avidity assay (DiaPROPH Med, Kiev, Ukraine; distributed by AlphaScience GmbH, Riedstadt, Germany; later also referred to as “DiaPROPH-Med antibody avidity assay”).

**Figure 3:**
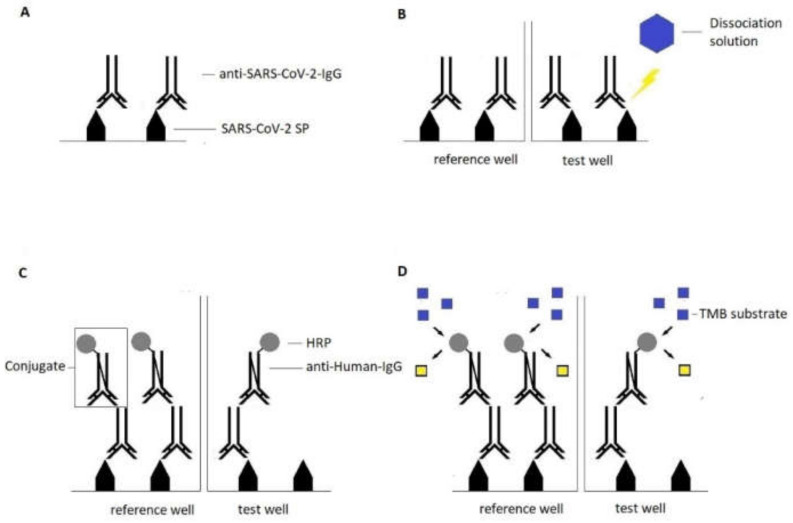
Principle of the DIA-SARS-CoV-2-S-IgG-av avidity assay. (**A**) Serum samples are added to the solid phase in duplicate (reference wells and test wells); present anti-SARS-CoV-2-RBD-IgG are bound to the antigen (recombinant SARS-CoV-2-spike protein) on the solid phase. (**B**) The test wells are treated with dissociation solution to destroy antibody-antigen complexes formed by low avidity antibodies. (**C**) Remaining antibody-antigen complexes are detected by the conjugate (HRP-labelled monoclonal anti-human-IgG). (**D**) Tetramethylbenzide substrate is added and reacts with HRP; the reference well indicates whether the sample contains a sufficient amount of IgG or not, if the first applies, the test well is used to calculate the RAI (Relative Avidity Index). Yellow square: color change after enzymatic reaction.

To determine a sample’s avidity, serum is placed in two wells simultaneously, hereafter referred to as the reference well and test well, respectively. In both wells, anti-SARS-CoV-2-IgG present in the sample are bound to immobilized recombinant SARS-CoV-2 spike protein on the solid phase. The test wells are then treated with a dissociation solution, which destroys low avidity antibody–antigen complexes formed in the first step. Remaining complexes in both wells are then detected by an HRP-labelled monoclonal anti-human IgG. TMB is added as the substrate for the enzymatic reaction. When read at 450/620 nm, the reference well’s OD is directly correlated with the concentration of present anti-SARS-CoV-2-IgG. The test well’s OD relative to the reference well’s OD is used to calculate the RAI (Relative Avidity Index). If the reference wells indicate quantitative IgG above a cut-off, results can be interpreted as low avidity (RAI < 40%) and high avidity (>40%). A schematic visualization is provided in Figure 3.

Measurement of the total IgG-antibodies directed against the receptor binding domain of the SARS-CoV-2 spike protein (anti-spike-RBD-IgG) were assessed applying the SARS-CoV-2-IgG-II-Quant assay on the Architect i2000SR analyzer (Abbott, North Chicago, IL, USA, later also referred to as “total S-antigen-specific anti-SARS-CoV-2-IgG assay”).

### 2.3. Tissue Culture Infecting Dose-(TCID-)Based Neutralization Assay

Serum samples that showed discrepant results in the surrogate neutralization assays were additionally tested with a tissue culture infecting dose (TCID)-based neutralization assay. Serum samples were serially diluted and incubated with 250 pfu/mL SARS-CoV-2 virus for 1h at room temperature. Antibody-virus mixtures were added to 96-well plates containing monolayers of Vero E6 cells (ATCC CRL-1586). Cells were cultured at 37 °C until cytopathic effect was observed. Neutralization titers were expressed as TCID. Titers ≤ 1:4 were considered negative.

### 2.4. Statistical Assessments

Both surrogate neutralization assays were tested against a composite reference standard consisting of their combined results. In detail, samples with concordantly positive results in both surrogate neutralization assays were considered as “truly” positive for neutralization antibodies, samples with concordantly negative results as “truly” negative. In case of discordant results of the composite reference standard, the TCID-(tissue culture infecting dose-)based neutralization assay was applied for confirmatory testing. Its results were considered as definite. Based on those assumptions, diagnostic accuracy values were calculated for both surrogate neutralization assays. To ensure hardest possible assessment conditions, “indetermined” results were counted as “negative” for the sensitivity assessment and as “positive” for the specificity assessment.

In addition, it was assessed whether different exposition types either to SARS-CoV-2 or to vaccination were associated with an increased or a decreased likelihood of expressing neutralizing antibodies. Further, the association of exposition types and antibody avidity values was assessed. Finally, correlation between titer levels of total S-antigen-specific anti-SARS-CoV-2 S-antibody titers on the one hand and antibody avidity values on the other hand with the levels of neutralizing antibodies as measured with both surrogate neutralization assays was assessed applying Spearman’s correlation coefficient.

Considering the low number of included samples, assessments were restricted to descriptive statistics and simple statistic approaches like Fisher’s exact test, Kruskal–Wallis rank sum testing and Wilcoxon rank sum testing.

## 3. Results

### 3.1. Diagnostic Accuracy for Both Surrogate Neutralization Assays

Based on the abovementioned assumptions on “true positivity” and “true negativity” for the surrogate neutralization assays, calculated sensitivity of the TECOmedical assay and the DiaPROPH-Med assay were 100% and 95.0%, respectively, while specificity was 61.3% and 48.4%, respectively. Details are provided in Table 1. In addition, the results of the Abbott total S-antigen-specific anti-SARS-CoV-2 IgG were given. For anti-spike IgG, a protective cut-off of 50 BAU/mL has been established by the World Health Organization (WHO). Cohen’s kappa (95% CI) between the gold standard used in this study, based on concordance of surrogate neutralization assays and TCID assay results against the protective BAU/mL is 0.440 (0.274–0.605). The sensitivity and specificity of the Abbott total S-antigen-specific anti-SARS-CoV-2 IgG protective BAU/mL with respect to the standard used here are 88.8 and 58.1, respectively.

### 3.2. Associations between Exposition Types and the Abundance of Neutralizing Antibodies Based on the Results of the Composite Reference Standard as well as Antibody Avidity and Correlation Assessments

Three types of exposition, i.e., one received vaccination, two received vaccinations, and natural SARS-CoV-2 infection were comparatively evaluated regarding an increased or a decreased likelihood of expressing neutralizing antibodies as well as with the levels of antibody avidity as measured with the DiaPROPH-Med avidity assay.

There was an association between exposition type and the overall likelihood of expressing neutralizing antibodies. While only 60 of 81 (74.07%) of the samples with only one vaccination showed an expression of neutralizing antibodies, this was the case for 85.71% (60 of 70 samples) of the samples from individuals with two vaccinations and 100% (40 of 40) of the samples from previously infected individuals. This association was statistically significant (Fisher’s exact test, *p* value < 0.001).

There was also a difference between the levels of antibody avidity as measured with the DiaPROPH-Med avidity assay for the samples from individuals with one and two vaccinations as well as from already infected individuals. Also, differences were seen for the association of exposition type and neutralizing antibodies as measured by the surrogate neutralization assays with best neutralizing effects after two vaccines. The details on the avidity levels and surrogate neutralization levels by exposition type are indicated in Table 2.

Because a normal distribution could not be assumed for the recorded avidity levels with Shapiro testing indicating *p* values of 0.0298, 0.00061, and <0.00001 for samples from individuals with one vaccination, 0.11161, <0.0001, and <0.00001 for samples from individuals with two vaccinations and 0.98165, 0.02723, and <0.00001 for samples from previously infected individuals, Kruskal–Wallis rank sum testing was conducted. Post-hoc conducted Wilcoxon rank sum testing indicated statistical significance for all two-group-comparisons (one vaccination vs. two vaccinations with *p* values < 0.0001, <0.0001, and 0.0002, one vaccination vs. COVID-19 infection with *p*-values of <0.0001, <0.0001, and 0.0055, two vaccination vs. COVID-19 infection with *p*-values of <0.0001 for all three tests).

Finally, correlation between anti-spike-RBD-IgG titers on the one hand and antibody avidity values on the other hand with the levels of neutralizing antibodies as measured with both surrogate neutralization assays was assessed using Spearman’s correlation coefficient. The measured coefficients ranged from 0.19 to 0.88. All pairwise correlations were positive, indicating that higher titers in one assay were associated with higher titers in the other assay. The *p*-values of all calculated correlation coefficients were less than 0.05. Details are provided in Table 3.

## 4. Discussion

The evaluation was conducted to comparably assess the diagnostic accuracy of two surrogate neutralization assays for the estimation of the neutralizing potential of anti-SARS-CoV-2 antibodies. The assessment confirmed good sensitivity of the TECOmedical assay, while its specificity scored worse than in previous assessments [24,25]. The DiaPROPH-Med surrogate neutralization test performed slightly worse in direct comparison in terms of its sensitivity and specificity, but has a more balanced price-performance ratio. Accordingly, respective diagnostic results should be interpreted with care. Of note, indetermined results as indicated by the DiaPROPH-Med surrogate neutralization assay were interpreted in the strictest possible way to ensure a thorough assessment. So, “indetermined” was interpreted like “negative” for the sensitivity assessment and like “positive” for the specificity assessment. However, the sensitivity of both surrogate neutralization assays is markedly higher than considering protective BAU/mL of quantitative total S-antigen-specific anti-SARS-CoV-2 IgG testing.

As a side finding, it could be shown with both assays that the abundance of neutralizing antibodies was increased in individuals after two vaccines as well as in individuals with natural infection compared to individuals with just one vaccine. This finding is not surprising, as vaccination trials have shown increased neutralizing potential after repeated vaccination [53]. For maturation-related avidity testing, the findings pointed in similar direction. Finally, correlation analysis of anti-spike-RBD-IgG-antibodies, neutralizing antibodies, and antibody avidity did not lead to conclusive associations allowing reliable predictions, although positive correlation was shown for the most parameters.

The evaluation has a number of limitations. First, only a moderate number of samples could be included in the assessment. Second, cell culture-based neutralization assays could only be run in case of contradicting results due to organizational reasons and funding restrictions.

## 5. Conclusions

The assessment confirmed acceptable diagnostic sensitivity for both surrogate neutralization assays. In contrast, further technical improvement of both assays seems advisable to increase their specificity and so, their results should be interpreted with care. Nevertheless, surrogate neutralization assays appear superior compared with the assessment of protective BAU/mL based on quantitative total S-antigen-specific anti-SARS-CoV-2 IgG detection.

## Figures and Tables

**Table 1:** Diagnostic accuracy as calculated for the assessed surrogate neutralization assays.

Assay	*n*	Overall Positives *n* (%)	Correct Positives (Sensitivity *n*% (with 0.95 CI))	Overall Negatives *n* (%)	Correct Negatives (Specificity *n*% (with 0.95 CI))	Indetermined *n* (%)
TECOmedical	191	172 (90.05)	160 (100%, (n.e.))	19 (9.95)	19 (61.29%, (43.34, 76.62))	0
DiaPROPH Med	191	166 (86.91)	152 (95.00%, (90.28, 97.49))	22 (11.52)	15 (48.39%, (31.57, 65.57))	3 (1.57)
Total S-antigen-specific anti-SARS-CoV-2-IgG (protective BAU/mL)	191	155 (81.15)	142 (88.75, (82.81, 92.82))	36 (18.85)	18 (58.06, (40.31, 73.95))	0

95% CI = 95% confidence interval. n.e. = not estimable. *n* = total numbers.

**Table 2:** Association of exposition types, antibody avidity as well as neutralizing effects of the measured antibodies.

Assay	Exposition Type	*n*	Mean (SD)	Median (Min, Max)	*p* Value
DiaPROPH-Med antibody avidity assay	One vaccination	81	33.58 (25.14)	30.84 (0, 96.83)	0.0001
	Two vaccinations	70	85.19 (11.14)	86.76 (59.24, 105.75)	
	Previously SARS-CoV-2 infected	40	58.28 (27.08)	58.70 (0, 117.40)	
TECOmedical surrogate neutralization assay	One vaccination	81	44.07 (27.63)	48.30 (0, 91.30)	0.0001
	Two vaccinations	70	98.27 (8.35)	99.70 (31.70, 99.90)	
	Previously SARS-CoV-2 infected	40	80.35 (14.71)	81.05 (44.30, 99.30)	
DiaPROPH-Med surrogate neutralization assay	One vaccination	81	77.60 (19.22)	81.67 (2.05, 96.26)	0.0001
	Two vaccinations	70	78.34 (31.33)	93.97 (0, 99.14)	
	Previously SARS-CoV-2 infected	40	72.28 (17.06)	75.23 (0, 94.91)	

SD = standard deviation. Min. = minimum. Max. = maximum. *n* = total numbers.

**Table 3:** Spearman’s correlation coefficient indicating correlation between levels of anti-spike-RBD-IgG titers on the one hand and antibody avidity values on the other hand with the levels of neutralizing antibodies as measured with both surrogate neutralization assays.

Spearman’s Rho N *p* Value	TECOmedical Surrogate Neutralization Assay	DiaPROPH-Med Surrogate Neutralization Assay	DiaPROPH-Med Antibody Avidity Assay	Total S-Antigen- Specific anti-SARS-CoV-2-IgG
TECOmedical surrogate neutralization assay	1			
DiaPROPH-Med surrogate neutralization assay	0.3101 191 <0.0001	1		
DiaPROPH-Med antibody avidity assay	0.6980	0.1859	1	
	191	191		
	<0.0001	0.0100		
Total S-antigen-specific anti-SARS-CoV-2-IgG	0.8844	0.2652	0.5790	1
	191	191	191	
	0.0001	0.0002	<0.0001	

## Data Availability

All relevant data are shown within the manuscript. Raw data can be provided at reasonable request.

## References

[1] Singanayagam A., Hakki S., Dunning J., Madon K.J., Crone M.A., Koycheva A., Derqui-Fernandez N., Barnett J.L., Whitfield M.G., Varro R. (2022). Community transmission and viral load kinetics of the SARS-CoV-2 delta (B.1.617.2) variant in vaccinated and unvaccinated individuals in the UK: A prospective, longitudinal, cohort study. Lancet Infect. Dis..

[2] Espejo A.P., Akgun Y., Al Mana A.F., Tjendra Y., Millan N.C., Gomez-Fernandez C., Cray C. (2020). Review of current advances in serologic testing for COVID-19. Am. J. Clin. Pathol..

[3] Gundlapalli A.V., Salerno R.M., Brooks J.T., Averhoff F., Petersen L.R., McDonald L.C., Iademarco M.F., CDC COVID-19 Response (2020). SARS-CoV-2 serologic assay needs for the next phase of the US COVID-19 pandemic response. Open Forum Infect. Dis..

[4] Emmerich P., Murawski C., Ehmen C., von Possel R., Pekarek N., Oestereich L., Duraffour S., Pahlmann M., Struck N., Eibach D. (2021). Limited specificity of commercially available SARS-CoV-2 IgG ELISAs in serum samples of African origin. Trop. Med. Int. Health.

[5] Sariol C.A., Pantoja P., Serrano-Collazo C., Rosa-Arocho T., Armina A., Cruz L., Stone E.T., Arana T., Climent C., Latoni G. (2021). Function is more reliable than quantity to follow up the humoral response to the receptor binding domain of SARS- CoV-2 spike protein after natural infection or COVID-19 vaccination. medRxiv.

[6] Oguntuyo K.Y., Stevens C.S., Hung C.T., Ikegame S., Acklin J.A., Kowdle S.S., Carmichael J.C., Chiu H.P., Azarm K.D., Haas G.D. (2021). Quantifying absolute neutralization titers against SARS-CoV-2 by a standardized virus neutralization assay allows for cross-cohort comparisons of COVID-19 sera. mBio.

[7] Pang N.Y., Pang A.S., Chow V.T., Wang D.Y. (2021). Understanding neutralising antibodies against SARS-CoV-2 and their implications in clinical practice. Mil. Med. Res..

[8] Jeewandara C., Jayathilaka D., Gomes L., Wijewickrama A., Narangoda E., Idampitiya D., Guruge D., Wijayamuni R., Manilgama S., Ogg G.S. (2021). SARS-CoV-2 neutralizing antibodies in patients with varying severity of acute COVID-19 illness. Sci. Rep..

[9] Chen W., Zhang J., Qin X., Wang W., Xu M., Wang L.F., Xu C., Tang S., Liu P., Zhang L. (2020). SARS-CoV-2 neutralizing antibody levels are correlated with severity of COVID-19 pneumonia. Biomed. Pharmacother..

[10] Bošnjak B., Stein S.C., Willenzon S., Cordes A.K., Puppe W., Bernhardt G., Ravens I., Ritter C., Schultze-Florey C.R., Gödecke N. (2021). Low serum neutralizing anti-SARS-CoV-2 S antibody levels in mildly affected COVID-19 convalescent patients revealed by two different detection methods. Cell. Mol. Immunol..

[11] Focosi D., Franchini M. (2021). Clinical predictors of SARS-CoV-2 neutralizing antibody titers in COVID-19 convalescents: Implications for convalescent plasma donor recruitment. Eur. J. Haematol..

[12] Mazzini L., Martinuzzi D., Hyseni I., Benincasa L., Molesti E., Casa E., Lapini G., Piu P., Trombetta C.M., Marchi S. (2021). Comparative analyses of SARS-CoV-2 binding (IgG, IgM, IgA) and neutralizing antibodies from human serum samples. J. Immunol. Methods.

[13] Kitagawa Y., Imai K., Matsuoka M., Fukada A., Kubota K., Sato M., Takada T., Noguchi S., Tarumoto N., Maesaki S. (2022). Evaluation of the correlation between the access SARS-CoV-2 IgM and IgG II antibody tests with the SARS-CoV-2 surrogate virus neutralization test. J. Med. Virol..

[14] Krone M., Gütling J., Wagener J., Lâm T.T., Schoen C., Vogel U., Stich A., Wedekink F., Wischhusen J., Kerkau T. (2021). Performance of three SARS-CoV-2 immunoassays, three rapid lateral flow tests, and a novel bead-based affinity surrogate test for the detection of SARS-CoV-2 antibodies in human serum. J. Clin. Microbiol..

[15] Olbrich L., Castelletti N., Schälte Y., Garí M., Pütz P., Bakuli A., Pritsch M., Kroidl I., Saathoff E., Guggenbuehl Noller J.M. (2021). Head-to-head evaluation of seven different seroassays including direct viral neutralisation in a representative cohort for SARS-CoV-2. J. Gen. Virol..

[16] Salazar E., Kuchipudi S.V., Christensen P.A., Eagar T., Yi X., Zhao P., Jin Z., Long S.W., Olsen R.J., Chen J. (2020). Convalescent plasma anti-SARS-CoV-2 spike protein ectodomain and receptor-binding domain IgG correlate with virus neutralization. J. Clin. Investig..

[17] Case J.B., Rothlauf P.W., Chen R.E., Liu Z., Zhao H., Kim A.S., Bloyet L.M., Zeng Q., Tahan S., Droit L. (2020). Neutralizing antibody and soluble ace2 inhibition of a replication-competent VSV-SARS-CoV-2 and a clinical isolate of SARS-CoV-2. Cell Host Microbe.

[18] Abe K.T., Li Z., Samson R., Samavarchi-Tehrani P., Valcourt E.J., Wood H., Budylowski P., Dupuis A.P., Girardin R.C., Rathod B. (2020). A simple protein-based surrogate neutralization assay for SARS-CoV-2. JCI Insight.

[19] Cameron A., Porterfield C.A., Byron L.D., Wang J., Pearson Z., Bohrhunter J.L., Cardillo A.B., Ryan-Muntz L., Sorensen R.A., Caserta M.T. (2021). A Multiplex microsphere IgG assay for SARS-CoV-2 using ACE2-mediated inhibition as a surrogate for neutralization. J. Clin. Microbiol..

[20] Chan K.H., Leung K.Y., Zhang R.R., Liu D., Fan Y., Chen H., Yuen K.Y., Hung I.F. (2021). Performance of a surrogate SARS-CoV-2-neutralizing antibody assay in natural infection and vaccination samples. Diagnostics.

[21] Embregts C.W.E., Verstrepen B., Langermans J.A.M., Böszörményi K.P., Sikkema R.S., de Vries R.D., Hoffmann D., Wernike K., Smit L.A.M., Zhao S. (2021). Evaluation of a multi-species SARS-CoV-2 surrogate virus neutralization test. One Health.

[22] Fischer B., Lichtenberg C., Müller L., Timm J., Fischer J., Knabbe C. (2021). A combined strategy to detect plasma samples reliably with high anti-SARS-CoV-2 neutralizing antibody titers in routine laboratories. J. Clin. Virol..

[23] Kamaladasa A., Gunasekara B., Jeewandara C., Jayathilaka D., Wijewickrama A., Guruge D., Wijayamuni R., Tan T.K., Ogg G.S., Townsend A. (2021). Comparison of two assays to detect IgG antibodies to the receptor binding domain of SARS-CoV-2 as a surrogate marker for assessing neutralizing antibodies in COVID-19 patients. Int. J. Infect. Dis..

[24] Kohmer N., Rühl C., Ciesek S., Rabenau H.F. (2021). Utility of different surrogate enzyme-linked immunosorbent assays (sELISAs) for detection of SARS-CoV-2 neutralizing antibodies. J. Clin. Med..

[25] Krüttgen A., Lauen M., Klingel H., Imöhl M., Kleines M. (2022). Two novel SARS-CoV-2 surrogate virus neutralization assays are suitable for assessing successful immunization with mRNA-1273. J. Virol. Methods.

[26] Meyer B., Reimerink J., Torriani G., Brouwer F., Godeke G.J., Yerly S., Hoogerwerf M., Vuilleumier N., Kaiser L., Eckerle I. (2020). Validation and clinical evaluation of a SARS-CoV-2 surrogate virus neutralisation test (sVNT). Emerg. Microbes Infect..

[27] Müller K., Girl P., von Buttlar H., Dobler G., Wölfel R. (2021). Comparison of two commercial surrogate ELISAs to detect a neutralising antibody response to SARS-CoV-2. J. Virol. Methods.

[28] Murray M.J., McIntosh M., Atkinson C., Mahungu T., Wright E., Chatterton W., Gandy M., Reeves M.B. (2021). Validation of a commercially available indirect assay for SARS-CoV-2 neutralising antibodies using a pseudotyped virus assay. J. Infect..

[29] Nandakumar V., Profaizer T., Lozier B.K., Elgort M.G., Larragoite E.T., Williams E.S.C.P., Solis-Leal A., Lopez J.B., Berges B.K., Planelles V. (2021). Evaluation of a Surrogate enzyme-linked immunosorbent assay-based severe acute respiratory syndrome coronavirus 2 (SARS-CoV-2) cPass neutralization antibody detection assay and correlation with immunoglobulin G commercial serology assays. Arch. Pathol. Lab. Med..

[30] Neerukonda S.N., Vassell R., Herrup R., Liu S., Wang T., Takeda K., Yang Y., Lin T.L., Wang W., Weiss C.D. (2021). Establishment of a well-characterized SARS-CoV-2 lentiviral pseudovirus neutralization assay using 293T cells with stable expression of ACE2 and TMPRSS2. PLoS ONE.

[31] Papenburg J., Cheng M.P., Corsini R., Caya C., Mendoza E., Manguiat K., Lindsay L.R., Wood H., Drebot M.A., Dibernardo A. (2021). Evaluation of a commercial culture-free neutralization antibody detection kit for severe acute respiratory syndrome-related Coronavirus-2 and comparison with an antireceptor-binding domain enzyme-linked immunosorbent assay. Open Forum Infect. Dis..

[32] Perera R.A.P.M., Ko R., Tsang O.T.Y., Hui D.S.C., Kwan M.Y.M., Brackman C.J., To E.M.W., Yen H.L., Leung K., Cheng S.M.S. (2021). Evaluation of a SARS-CoV-2 surrogate virus neutralization test for detection of antibody in human, canine, cat, and hamster sera. J. Clin. Microbiol..

[33] Sancilio A.E., D’Aquila R.T., McNally E.M., Velez M.P., Ison M.G., Demonbreun A.R., McDade T.W. (2021). A surrogate virus neutralization test to quantify antibody-mediated inhibition of SARS-CoV-2 in finger stick dried blood spot samples. Sci. Rep..

[34] Schmidt F., Weisblum Y., Muecksch F., Hoffmann H.H., Michailidis E., Lorenzi J.C.C., Mendoza P., Rutkowska M., Bednarski E., Gaebler C. (2020). Measuring SARS-CoV-2 neutralizing antibody activity using pseudotyped and chimeric viruses. J. Exp. Med..

[35] Schuh W., Baus L., Steinmetz T., Schulz S.R., Weckwerth L., Roth E., Hauke M., Krause S., Morhart P., Rauh M. (2021). A surrogate cell-based SARS-CoV-2 spike blocking assay. Eur. J. Immunol..

[36] Sholukh A.M., Fiore-Gartland A., Ford E.S., Miner M.D., Hou Y.J., Tse L.V., Kaiser H., Zhu H., Lu J., Madarampalli B. (2021). Evaluation of cell-based and surrogate SARS-CoV-2 neutralization assays. J. Clin. Microbiol..

[37] Taylor S.C., Hurst B., Martiszus I., Hausman M.S., Sarwat S., Schapiro J.M., Rowell S., Lituev A. (2021). Semi-quantitative, high throughput analysis of SARS-CoV-2 neutralizing antibodies: Measuring the level and duration of immune response antibodies post infection/vaccination. Vaccine.

[38] Tiwari A.K., Negi G., Jaiswal R.M., Aggarwal G., Yadav N., Kumar V., Kulathu K. (2021). Correlation of sample-to-cut-off ratio of anti-SARS-CoV-2 IgG antibody chemiluminescent assay with neutralization activity: A prospective multi-centric study in India. ISBT Sci. Ser..

[39] Valcourt E.J., Manguiat K., Robinson A., Chen J.C., Dimitrova K., Philipson C., Lamoureux L., McLachlan E., Schiffman Z., Drebot M.A. (2021). Evaluation of a commercially-available surrogate virus neutralization test for severe acute respiratory syndrome coronavirus-2 (SARS-CoV-2). Diagn. Microbiol. Infect. Dis..

[40] Valcourt E.J., Manguiat K., Robinson A., Lin Y.C., Abe K.T., Mubareka S., Shigayeva A., Zhong Z., Girardin R.C., DuPuis A. (2021). Evaluating humoral immunity against SARS-CoV-2: Validation of a plaque-reduction neutralization test and a multilaboratory comparison of conventional and surrogate neutralization assays. Microbiol. Spectr..

[41] Vandergaast R., Carey T., Reiter S., Lathrum C., Lech P., Gnanadurai C., Haselton M., Buehler J., Narjari R., Schnebeck L. (2021). IMMUNO-COV v2.0: Development and validation of a high-throughput clinical assay for measuring SARS-CoV-2-Neutralizing antibody titers. mSphere.

[42] Von Rhein C., Scholz T., Henss L., Kronstein-Wiedemann R., Schwarz T., Rodionov R.N., Corman V.M., Tonn T., Schnierle B.S. (2021). Comparison of potency assays to assess SARS-CoV-2 neutralizing antibody capacity in COVID-19 convalescent plasma. J. Virol. Methods.

[43] Wagner T.R., Ostertag E., Kaiser P.D., Gramlich M., Ruetalo N., Junker D., Haering J., Traenkle B., Becker M., Dulovic A. (2021). NeutrobodyPlex-monitoring SARS-CoV-2 neutralizing immune responses using nanobodies. EMBO Rep..

[44] Walker S.N., Chokkalingam N., Reuschel E.L., Purwar M., Xu Z., Gary E.N., Kim K.Y., Helble M., Schultheis K., Walters J. (2020). SARS-CoV-2 assays to detect functional antibody responses that block ACE2 recognition in vaccinated animals and infected patients. J. Clin. Microbiol..

[45] Wohlgemuth N., Whitt K., Cherry S., Kirkpatrick Roubidoux E., Lin C.Y., Allison K.J., Gowen A., Freiden P., Allen E.K., St Jude Investigative Team (2021). An Assessment of Serological Assays for SARS-CoV-2 as Surrogates for Authentic Virus Neutralization. Microbiol. Spectr..

[46] Jeong S., Lee N., Lee S.K., Cho E.J., Hyun J., Park M.J., Song W., Jung E.J., Woo H., Seo Y.B. (2021). Comparing results of five SARS-CoV-2 antibody assays before and after the first dose of ChAdOx1 nCoV-19 vaccine among health care workers. J. Clin. Microbiol..

[47] Jeong S., Lee N., Lee S.K., Cho E.J., Hyun J., Park M.J., Song W., Jung E.J., Woo H., Seo Y.B. (2021). Comparison of the results of five SARS-CoV-2 antibody assays before and after the first and second ChAdOx1 nCoV-19 vaccinations among health care workers: A prospective multicenter study. J. Clin. Microbiol..

[48] McDade T.W., Demonbreun A.R., Sancilio A., Mustanski B., D’Aquila R.T., McNally E.M. (2021). Durability of antibody response to vaccination and surrogate neutralization of emerging variants based on SARS-CoV-2 exposure history. Sci. Rep..

[49] Sholukh A.M., Fiore-Gartland A., Ford E.S., Hou Y., Tse L.V., Lempp F.A., Kaiser H., Saint Germain R., Bossard E., Kee J.J. (2020). Evaluation of SARS-CoV-2 neutralization assays for antibody monitoring in natural infection and vaccine trials. medRxiv.

[50] Yin Q., Zhang Y., Lian L., Qu Y., Wu W., Chen Z., Pei R., Chen T., Sun L., Li C. (2021). Chemiluminescence immunoassay based serological immunoassays for detection of SARS-CoV-2 neutralizing antibodies in COVID-19 convalescent patients and vaccinated population. Viruses.

[51] Pichler D., Baumgartner M., Kimpel J., Rössler A., Riepler L., Bates K., Fleischer V., von Laer D., Borena W., Würzner R. (2021). Marked increase in avidity of SARS-CoV-2 antibodies 7–8 months after infection is not diminished in old age. J. Infect. Dis..

[52] Hollstein M.M., Münsterkötter L., Schön M.P., Bergmann A., Husar T.H., Abratis A., Eidizadeh A., Schaffrinski M., Zachmann K., Schmitz A. Interdependencies of Cellular and Humoral Immune Responses in Heterologous and Homologous SARS-CoV-2 Vaccination. https://www.medrxiv.org/content/10.1101/2021.12.13.21267729v1.

[53] Walsh E.E., Frenck R.W., Falsey A.R., Kitchin N., Absalon J., Gurtman A., Lockhart S., Neuzil K., Mulligan M.J., Bailey R. (2020). Safety and immunogenicity of two RNA-based COVID-19 vaccine candidates. N. Engl. J. Med..

